# Increased Ascorbate Biosynthesis Does Not Improve Nitrogen Fixation Nor Alleviate the Effect of Drought Stress in Nodulated *Medicago truncatula* Plants

**DOI:** 10.3389/fpls.2021.686075

**Published:** 2021-06-28

**Authors:** Libertad Cobos-Porras, María Isabel Rubia, Raúl Huertas, David Kum, David A. Dalton, Michael K. Udvardi, Cesar Arrese-Igor, Estíbaliz Larrainzar

**Affiliations:** ^1^Institute for Multidisciplinary Applied Biology (IMAB), Universidad Pública de Navarra (UPNA), Pamplona, Spain; ^2^Plant Biology Division, Noble Research Institute LLC, Ardmore, OK, United States; ^3^Biology Department, Reed College, Portland, OR, United States

**Keywords:** ascorbic acid, legume, symbiosis, water deficit, antioxidant

## Abstract

Legume plants are able to establish nitrogen-fixing symbiotic relations with *Rhizobium* bacteria. This symbiosis is, however, affected by a number of abiotic constraints, particularly drought. One of the consequences of drought stress is the overproduction of reactive oxygen (ROS) and nitrogen species (RNS), leading to cellular damage and, ultimately, cell death. Ascorbic acid (AsA), also known as vitamin C, is one of the antioxidant compounds that plants synthesize to counteract this oxidative damage. One promising strategy for the improvement of plant growth and symbiotic performance under drought stress is the overproduction of AsA via the overexpression of enzymes in the Smirnoff-Wheeler biosynthesis pathway. In the current work, we generated *Medicago truncatula* plants with increased AsA biosynthesis by overexpressing *MtVTC2*, a gene coding for GDP-L-galactose phosphorylase. We characterized the growth and physiological responses of symbiotic plants both under well-watered conditions and during a progressive water deficit. Results show that increased AsA availability did not provide an advantage in terms of plant growth or symbiotic performance either under well-watered conditions or in response to drought.

## Introduction

Legumes are able to establish nitrogen-fixing symbioses with soil bacteria of the Rhizobiaceae family. This interaction provides a source of nitrogen for plant growth and reproduction, reducing the need for synthetic fertilizers in agricultural systems. However, establishment and functioning of these symbioses is negatively affected by a number of stresses. Among these, water deficit inhibits symbiotic nitrogen fixation (SNF) in both temperate and tropical legumes, although the molecular and physiological bases remain unclear (Serraj et al., 2001; King and Purcell, 2005; Marino et al., 2007; Gil-Quintana et al., 2013a,b). One of the effects of drought stress in plants generally is an overproduction of reactive oxygen (ROS) and nitrogen species (RNS), leading to oxidative damage at the cellular level and, ultimately, cell death (Smirnoff, 1993; Moran et al., 1994; Cruz De Carvalho, 2008). Plants are able to counteract such damage via the production of antioxidant compounds such as ascorbic acid (AsA). AsA, also known as vitamin C, is a water-soluble vitamin essential for plant and animal growth and development. In plants, AsA is involved in a number of physiological processes including photosynthesis, cell wall growth, seed germination, flowering time, and senescence, among others (Foyer and Noctor, 2011; Zechmann, 2011; Mellidou et al., 2012; Tóth et al., 2013). Additionally, it is the substrate of ascorbate peroxidase within the AsA-glutathione (AsA-GSH) or Foyer-Halliwell-Asada pathway (Foyer and Noctor, 2011), which mitigates ROS and RNS damage in plant cells.

The AsA-GSH cycle is important for the maintenance of SNF in root nodules (Matamoros et al., 2003; Becana et al., 2010). Nodule tissue is enriched in the enzymes of the AsA-GSH cycle, and the activities of these enzymes are strongly correlated with nodule effectiveness (Dalton et al., 1993, 1998). A current hypothesis is that increased AsA pools under stressful conditions should lead to improved (or at least less affected) SNF. Indeed, stem infusion of 10 mM AsA has been shown to increase nitrogenase activity 4-fold (based on acetylene reduction measurements) in soybean plants (Bashor and Dalton, 1999). Similarly, *in vitro* assays using isolated rhizobia, myoglobin/leghemoglobin, 2 mM AsA, and recombinant AsA peroxidase led a significant increase in nitrogenase activity (Ross et al., 1999). Despite these promising results, a formal *in planta* approach to increase the levels of AsA in nitrogen-fixing legume plants has not been undertaken so far.

Although alternative pathways have been described, the Smirnoff-Wheeler or D-mannose/L-galactose pathway is considered the primary route of AsA biosynthesis in plants (Wheeler et al., 1998; Conklin et al., 1999; Dowdle et al., 2007). The rate-limiting and committed step for AsA biosynthesis is catalyzed by GDP-L-galactose phosphorylase, which is encoded by the genes *AtVTC2* and *AtVTC5* in the model plant, *A. thaliana* (Laing et al., 2007; Linster et al., 2007; Smirnoff, 2007). Double mutants in *AtVTC2* and *AtVTC5* were unable to grow unless supplemented with AsA (Dowdle et al., 2007), demonstrating the key role of these enzymes in the biosynthesis pathway.

In the current work, we tested the hypothesis that increased AsA in nodules can lead to improved symbiotic performance in the model legume, *Medicago truncatula*. We generated stably-transformed *M. truncatula* plants with increased AsA biosynthesis by overexpressing the *MtVTC2* gene and characterized the growth of plants in symbiosis with *Ensifer* (*Sinorhizobium*) *meliloti* bacteria under well-watered conditions and during progressive water deficit. Results show that higher levels of AsA in nodules did not improve symbiosis establishment/nodule functioning or ameliorate the effects of drought stress on plant performance.

## Materials and Methods

### Biological Material

To construct *Medicago truncatula* lines overexpressing *MtVTC2*, the coding sequence of the gene Medtr5g093390.1 (including the stop codon) was amplified from a *M. truncatula* Jemalong A17 leaf cDNA library using Phusion High-Fidelity DNA Polymerase (Thermo Fisher Scientific Inc.). Primers 5′-CACCATGATGCTAAAAATCAAAAGGG-3′ and 5′-TTACTGTAGAACAACACATTCTTGTGAAC-3′ were used for directional cloning into pENTR/D-TOPO (Life Technologies). Sequence-verified constructs were recombined into the destination binary vector pK7RWG2 (https://gatewayvectors.vib.be/) with LR clonase (Invitrogen) and transformed into One Shot TOP10 chemically competent *E. coli* (Invitrogen). The empty vector pK7RWG2, lacking the *MtVTC2* gene, was employed as a control. Sequence-verified binary vectors were transformed into *Agrobacterium tumefaciens* strain EHA105 and 10 stable transgenic *M. truncatula* R108 plants per construct were obtained by *A. tumefaciens*-mediated transformation as previously described (Jiang et al., 2019).

Two microsymbionts were used: *Ensifer (Sinorhizobium) meliloti* strain 2011 constitutively expressing either *LacZ* (pXLGD4; Ardourel et al., 1994) or GFP, which are classical reporter genes to monitor bacteroids in nodules. Bacteria were grown at 28°C for 48 h in yeast extract mannitol broth containing (g L^−1^): mannitol (10), yeast extract (0.4), NaCl (0.1), K_2_HPO_4_ (0.5), 0.2 MgSO_4_·7H_2_O (Vincent, 1970). Seedlings were inoculated twice with 1 mL of the corresponding strain the day of planting and 1 week afterwards (corresponding to ~1 × 10^9^ colony forming units mL^−1^).

### Growth Conditions and Drought Treatment

*M. truncatula* R108 seeds of the empty vector and overexpressing lines were grown in 1-L pots with a mixture of vermiculite:perlite (5:2, v/v) as substrate under controlled environmental conditions (14-h day/10-h night; 600 μmol m^−2^ s^−1^ light intensity; 22/16°C day/night temperature; 70–60% relative humidity). Plants were inoculated as described above and watered with nutrient solution (Evans, 1981) containing 0.25 mM ammonium nitrate for the first 2 weeks. The nutrient solution remained N-free afterwards. Two-month-old plants (i.e., 8 weeks post-inoculation) from each line were separated into three sets according to their values of leaf water potential (Ψ_leaf_). One set of plants (control, C) was maintained to field capacity and the two others were subjected to water deprivation for the establishment of drought stress (mild and severe drought, MD and SD, respectively). To establish the effect of water deficit on plants, transpiration rates, stomatal conductance, leaf and nodule water potentials were measured. Plant transpiration rates were gravimetrically determined daily on a whole-plant basis. Stomatal conductance was measured with an AP4 porometer (Delta-T Devices). Ψ_leaf_ was estimated using a Scholander pressure chamber (Soil Moisture Equipment). Stomatal conductance, Ψ_leaf_ and chlorophyll content were determined in the youngest fully expanded leaf. Nodule water potential (Ψ_nodule_) was analyzed using C52 sample chambers coupled to a Wescor HR-33T Dew Point Microvoltmeter (Wescor). The relative levels of leaf chlorophyll were estimated using a portable SPAD-502 meter (Soil Plant Analysis Development, Konica-Minolta Inc.). Symbiotic nitrogen fixation was estimated as apparent nitrogenase activity (ANA) according to the method described by Witty and Minchin (1998). H_2_ evolution of intact plants was measured in an open flow-through system under N_2_/O_2_ (79/21%) using an electrochemical H_2_ sensor (Qubit System Inc.). The H_2_ sensor was calibrated with high purity gases (Praxair) using a gas mixer (Air Liquide) flowing at the same rate as the sampling system (500 mL min^−1^).

Plants were physiological characterized, and then, leaf, root and nodule samples were collected and frozen in liquid N_2_ and stored at −80°C for further analysis. Other set of samples were weighed for fresh weight (FW) determinations and, subsequently, oven-dried at 70°C for 48 h before dry weight (DW) determinations.

### *MtVTC2* Gene Identification and Phylogenetic Analysis

The putative *VTC2* and *VTC5* orthologous genes in *M. truncatula* and other plant species were identified by BLASTP (cutoff E-value <1E^−10^, identity >60%) using the *A. thaliana* proteins VTC2 (AT4G26850.1) and VTC5 (AT5G55120.1) as baits. Subsequently, a HMMER analysis was carried out to further validate the selection of candidates. Both analyses were performed in the Ensembl Plants website (http://plants.ensembl.org/) using default parameters, except for the *Lotus japonicus* sequences, which were analyzed independently using the Lotus Base website (https://lotus.au.dk/). The predicted amino acid sequences were aligned using MAFFT (http://mafft.cbrc.jp/alignment/server) defining G-INS-i as iterative refinement method and BLOSUM30 as scoring matrix. Phylogenetic analyses were conducted in MEGA7 (Kumar et al., 2016) using the Maximum Likelihood method based on the JTT amino acid substitution model and 1,000 bootstrap replications as previously described (Seminario et al., 2017).

### Determination of Total and Reduced Ascorbic Acid Content

Nodule and leaf AsA levels were measured by high-performance capillary electrophoresis, as previously described (Davey et al., 1996; Seminario et al., 2017). To obtain total AsA pools, samples were treated with dithiothreitol, and DHA levels were calculated as the difference between the total AsA pool and the reduced AsA levels.

### Determination of Sucrose Content

Nodule extracts were prepared from 0.1 g FW of nodules, as previously described (Marino et al., 2006). Sucrose was determined by ion-exchange chromatography in a 940 Professional IC Vario with amperometric detector (Metrohm AG) equipped with Metrosep Carb 2 Guard/4.0 + Metrosep Carb 2–150/4.0 columns. Samples were eluted with 300 mM NaOH/1 mM C_2_H_3_NaO_2_ at 30°C.

### RNA Extraction, Reverse Transcription, and qPCR Analysis

For RNA extraction, nodules were collected from roots of the *M. truncatula* EV and oxVTC2 lines 6 weeks post-inoculation and total RNA was extracted in 200 μL GTC lysis buffer (Omega Bio-Tek) containing 4 μL of β-mercaptoethanol. The lysate was then passed through a homogenizer column (Omega Bio-Tek) by centrifugation at 15,000 g and the flow-through was mixed with one volume of 100% ethanol. Nucleic acids were recovered following the Clean and Concentrator-5 protocol (Zymo Research) beginning at step 3 and eluted with 50 μL of nuclease-free water. The solution was treated with Turbo DNase (Invitrogen) to yield purified RNA following the manufacturer's instructions. RNA concentration and quality were measured using the Qubit RNA Broad Range assay (LifeTechnologies). RNA samples were further processed with at least RNA Quality Number values above 6.0 and 28S/18S area ratio values above 0.8. Single stranded cDNA was generated by reverse transcription using SuperScript IV Reverse Transcriptase (LifeTechnologies). Real-time quantitative PCR (qPCR) was performed on a Bio-Rad CFX96 System (Bio-Rad). The PCR reaction consisted of 7.5 μL of GoTaq qPCR Master Mix (Promega), 0.5 μM of forward and reverse primers, and 2.0 μl of template cDNA in a total volume of 15 μl. For the reference gene, the polypyrimidine tract-binding-like gene (PTB, Medtr3g090960) was used, a gene shown to present high transcript stability for *Medicago truncatula* samples at several developmental stages (Kakar et al., 2008). All reactions were set up in duplicate and three biological samples per genotype were analyzed. The PCR program consisted of a polymerase activation step of 2 min at 95°C, followed by 40 cycles of 15 s at 95°C and 30 s at 60°C. A final melting curve was included to confirm the specificity of the reaction. The software LinRegPCR (Ramakers et al., 2003; Ruijter et al., 2009) was employed to calculate the quantification cycles (Cq) and amplification efficiency of each primer pair. Relative quantification of *MtVTC2* transcripts was estimated using the 2^−ΔΔCq^ method (Livak and Schmittgen, 2001) since the differences in primer efficiency were <5%. A list of the primers used can be found as Supplementary Table 1.

### Microscopy

Plant nodules were harvested and cut with a vibratome (Lancer Series 1000) in 75 μm-longitudinal sections. The *in-situ* detection of H_2_O_2_ and ${\text{O}}_{2}^{-}$ in nodules was performed as described in Muglia et al. (2008). In plants inoculated with the LacZ-expressing *E. meliloti* strain, β-galactosidase activity in nodule sections was measured as previously described (Larrainzar et al., 2015). Nodule preparations were visualized with a Zeiss Axioskop2 optical microscope (Zeiss Microscopy) or a Leica fluorescence microscope DM750 (Leica).

### β-Galactosidase Activity in Bacteroids

The nodule bacteroid fraction was isolated as described by Saalbach et al. (2002). Briefly, 0.1 g FW of nodules were homogenized using a mortar and pestle in 1 ml of ice-cold buffer containing 50 mM HEPES (pH 7.5), 450 mM mannitol, 7 mM ethylenediaminotetraacetic acid (EDTA), 7 mM CaCl_2_, 5 mM MgCl_2_, and 5 mM di-thiothreitol (DTT). The mixture was filtered through Miracloth and centrifuged at 500 g for 10 min to remove cell debris. The supernatant was recovered and centrifuged again at 6000 g for 10 min to pellet the bacteroid fraction. The supernatant was discarded and the pellet was washed twice with extraction buffer and centrifuged at 10000 g for 10 min. β-galactosidase activity was measured based on the assay described by Miller (1972), subsequently adapted to a 96-well plate format (Griffith and Wolf, 2002). The bacteroid-containing pellet was resuspended in 1 mL of buffer Z. To permeabilize the bacteroids, 20 μL of 0.1% SDS and 40 μL of chloroform were added. The assay was initiated by adding 20 μL of orthonitrophenyl-β-D-galactopyranoside (ONPG; 4 mg/ml) and the time was noted. As soon as the yellow chromophore ortho-nitrophenol (ONP) was formed, the reaction was terminated by the addition of 50 μL of 1 M Na_2_CO_3_. Microplate readings were then determined at 420 and 550 nm, respectively. The β-galactosidase specific activities in Miller units are calculated based on the protein content of the bacteroid samples (Brito et al., 2008).

### Statistical Analysis

Results correspond to two independent experiments with a number of biological replicates for each ranging from 3 to 5. Experiments were arranged in a completely randomized design with 5 plants per treatment. The data were subjected to an analysis of two-way ANOVA with plant line as one factor and drought treatment as the other using the SPSS Statistics for Windows, version 24 (Armonk, NY, IBM corp.). Significant differences between treatments were determined by LSD (*p* ≤ 0.05) and are represented with different letters.

## Results

### Identification and Expression Profile of Genes Coding for GDP-L-galactose Phosphorylases in *M. truncatula*

**Figure 1:**
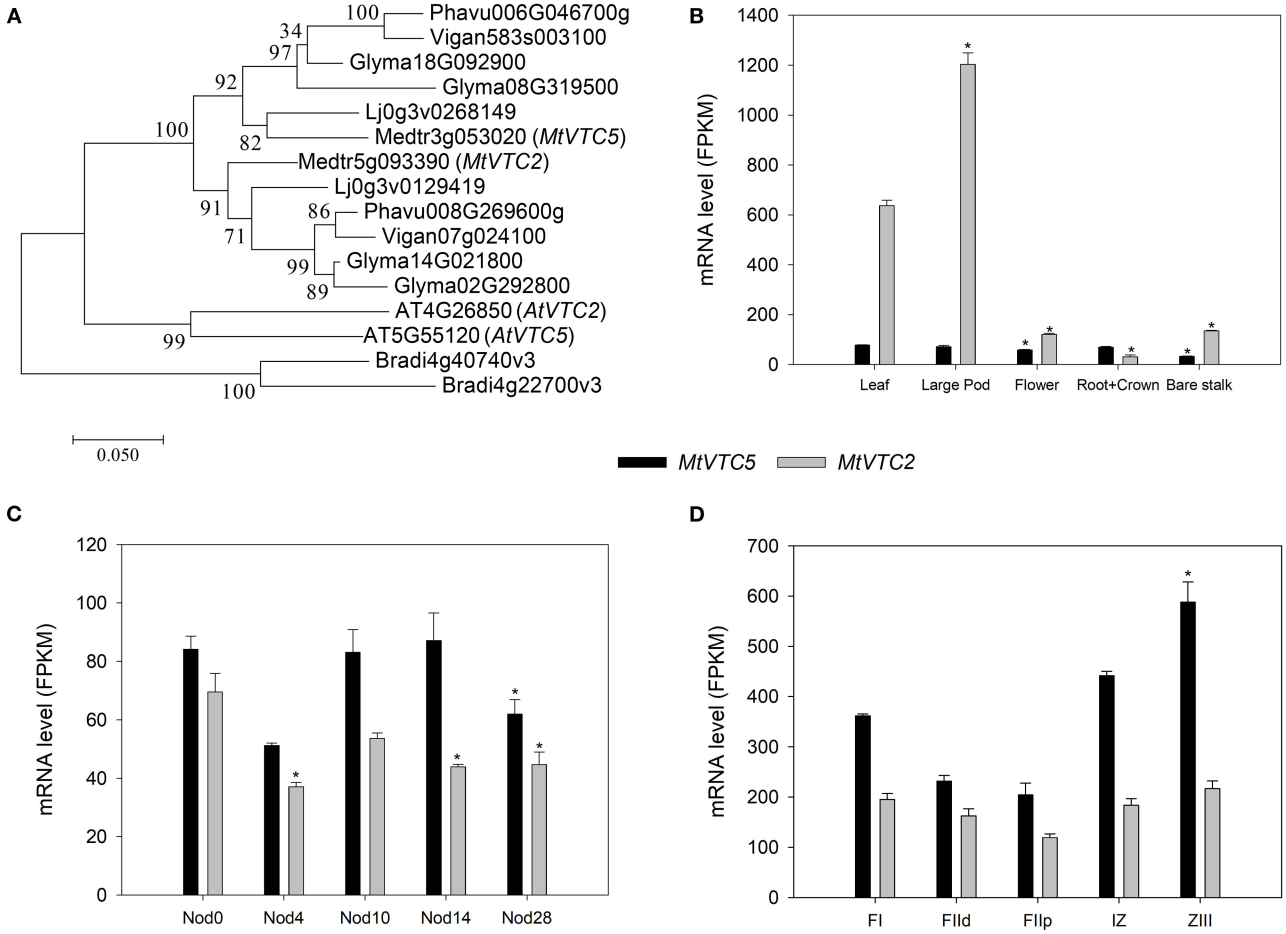
Phylogenetic analysis **(A)** and RNA-sequencing (RNA-seq) expression data corresponding to the *MtVTC2* and *MtVTC5* genes. The Maximum Likelihood method was applied to generate a phylogenetic tree (1,000 bootstrap replications) using the software Mega 7 (Kumar et al., 2016). Putative orthologs in *A. thaliana, Brachypodium distachyon, Glycine max, Phaseolus vulgaris*, and *Vigna angularis* were retrieved after BLASTP and HMMER analysis at the Ensembl Plants website (http://plants.ensembl.org/). Sequences corresponding to *Lotus japonicus* were extracted from the Lotus Base using BLASTP (https://lotus.au.dk/blast/) against the *Lotus japonicus* MG20 v3.0 Proteins database. Data corresponding to gene expression in different plant organs **(B)**, during nodule development **(C)**, and regions of a nitrogen-fixing nodule (**D**; FI: apical region; FIId and FIIp: distal and a proximal FII fraction, respectively; IZ: interzone; ZIII: nitrogen-fixation zone) was collected from the *M. truncatula* Small Secreted Peptide database (MtSSPdb; Boschiero et al., 2020; https://mtsspdb.noble.org/database) and Symbimics website (Roux et al., 2014; https://iant.toulouse.inra.fr/symbimics). Values represent the average ± SE (*n* = 3 biological replicates). FPKM, Fragments Per Kilobase Million. Asterisks denote significant differences (*p* ≤ 0.05) using ANOVA test with respect to the sample used as a reference: “Leaf” for panel **(B)**, “Nod0” for panel **(C)**, and FI region for panel **(D)**.

One of the key steps controlling AsA biosynthesis in plants is the conversion of GDP-L-galactose to L-galactose 1-P by GDP-L-galactose phosphorylase (EC 2.7.7.69). In *A. thaliana* two genes have been identified coding for this enzyme, *AtVTC2* (AT4G26850.1) and *AtVTC5* (AT5G55120.1; Dowdle et al., 2007). Using the predicted amino acid sequences of these two enzymes, BLASTp search of the *M. truncatula* genome retrieved the genes Medtr5g093390 (MtrunA17Chr5g0444591 in v5 of the genome; Pecrix et al., 2018) and Medtr3g053020 (MtrunA17Chr3g0099501) as the putative orthologs for *AtVTC2* and *AtVTC5*, respectively. We also performed reciprocal BLASTP followed by HMMER to retrieve additional orthologs within the legume family and built a phylogenetic tree using *Brachypodium distachyon* as outgroup (Figure 1A). Subsequently, we designated Medtr5g093390 as *MtVTC2* and Medtr3g053020 as *MtVTC5*.

We queried the *M. truncatula* Small Secreted Peptide database (MtSSPdb; Boschiero et al., 2020; https://mtsspdb.noble.org/database) to obtain expression profiles of these two genes in different *M. truncatula* organs and upon inoculation with symbiotic bacteria (Figures 1B–D). *MtVTC2* showed the highest relative expression in the different plant tissues measured, being particularly abundant in leaves and large pods (Figure 1B). In uninoculated roots, both *MtVTC2* and *MtVTC5* exhibited similar expression levels and neither showed a symbiosis-related increase in gene expression, even in mature nitrogen-fixing nodules at day 28 after inoculation (Figure 1C). Interestingly, fertilization of plants with nitrate, a classical inhibitor of nitrogen fixing activity, did not significantly alter the expression levels of these genes (data not shown). Looking at gene expression profiles in microdissected nodules in the Symbimics database (Roux et al., 2014; https://iant.toulouse.inra.fr/symbimics), both *MtVTC2* and *MtVTC*5 were present in all the nodule regions, with *MtVTC5* expression significantly higher in the nitrogen-fixing zone (ZIII), using the meristematic zone (FI) as a reference (Figure 1D). However, in terms of response to abiotic stresses, the expression profile of *MtVTC2* showed the most interesting pattern, with a strong induction in roots of *M. truncatula* subjected to a progressive drought stress (Seminario et al., 2017). Based on this expression analysis, we selected *MtVTC2* as a candidate to be overexpressed in nodules to test for improved nitrogen fixation and drought resilience.

### Overexpression of *MtVTC2* Increased the Total AsA Pool but Did Not Promote Plant Growth of Nitrogen-Fixing *M. truncatula* Under Optimal Water Conditions

To test if increased AsA in nodules could lead to an improved symbiosis or reduce the impact of drought stress, we generated stably transformed *M. truncatula* plants expressing *MtVTC2* under the control of the 35S promoter. From the several lines generated, we selected two independent lines per construct for further studies; lines oxVTC2#2-1 and oxVTC2#4 for *MtVTC2* overexpression, and lines EV#4 and EV#6 transformed with the empty vector (EV) as controls. To test the effect of the overexpression of *MtVTC2* in the AsA biosynthesis pathway, the levels of AsA, the total AsA pool and the ratio AsA/total AsA pool were measured in both leaves and nitrogen-fixing root nodules (Table 1). In leaf tissue, the oxVTC2 lines presented an average increase of +31% content of AsA compared to the EV lines. In nodules, results show that oxVTC2 lines presented an average increase of +51% content of AsA and +41% of the total AsA pool relative to the EV lines. Accordingly, qPCR analysis showed that the levels of expression of the *MtVTC2* in nodules of the oxVTC2 lines was 3.8-fold this of EV plants (Supplementary Figure 1).

**Table 1:** Analysis of the levels of ascorbic acid (AsA), total AsA pool, and AsA to total AsA pool ratios in leaves and nodules of *M. truncatula* empty vector (EV) and *MtVTC2* overexpression lines (oxVTC2) under well-watered conditions.

**Plant line**	**Leaves**			**Nodules**		
	**AsA (μmol g^**−1**^ LDW)**	**Total AsA (μmol g^**−1**^ LDW)**	**AsA/Total AsA**	**AsA (μmol g^**−1**^ NDW)**	**Total AsA (μmol g^**−1**^ NDW)**	**AsA/Total AsA**
EV	33.25 ± 1.71	48.87 ± 3.93	0.71 ± 0.07	7.81 ± 0.94	16.56 ± 1.73	0.49 ± 0.05
oxVTC2	43.53 ± 0.54*	49.20 ± 1.39	0.89 ± 0.02*	11.84 ± 0.94*	23.52 ± 2.87*	0.54 ± 0.04

Values represent the average ± SE (n = 4 biological replicates). Asterisks (*) denote significant differences (p ≤ 0.05) using ANOVA test. LDW, Leaf dry weight; NDW, nodule dry weight.

Regarding plant performance and symbiotic efficiency, under well-watered conditions both the overexpression and EV lines showed similar growth rates, with average total plant biomass of 2.04 ± 0.15 g DW and 2.19 ± 0.25 g DW, respectively (Table 2). Similarly, we did not observe significant differences in terms of nodule formation, and both sets of plants presented similar relative chlorophyll levels. We also analyzed the levels of nitrogen fixation, measured as apparent nitrogenase activity (ANA) in intact plants, and results show no statistical differences compared to the control lines (Table 2).

**Table 2:** Physiological characterization of *M. truncatula* empty vector (EV) and *MtVTC2* overexpression lines (oxVTC2) under well-watered conditions.

					**ANA**
**Plant line**	**SDW (g)**	**RDW (g)**	**NDW (g)**	**SPAD**	**(μmol H_**2**_ min^**−1**^ g^**−1**^ NDW)**
EV	1.29 ± 0.07	0.72 ± 0.08	0.034 ± 0.003	47.07 ± 0.44	4.85 ± 0.49
oxVTC2	1.46 ± 0.15	0.69 ± 0.08	0.039 ± 0.003	46.43 ± 1.18	5.15 ± 0.44

Plant biomass (SDW, shoot dry weight; RDW, root dry weight; NDW, nodule dry weight), relative chlorophyll content (estimated as SPAD units) and apparent nitrogenase activity (ANA). Values represent average ± SE (n = 8 biological replicates). Asterisks (*) denote significant differences (p ≤ 0.05, ANOVA test).

**Figure 2:**
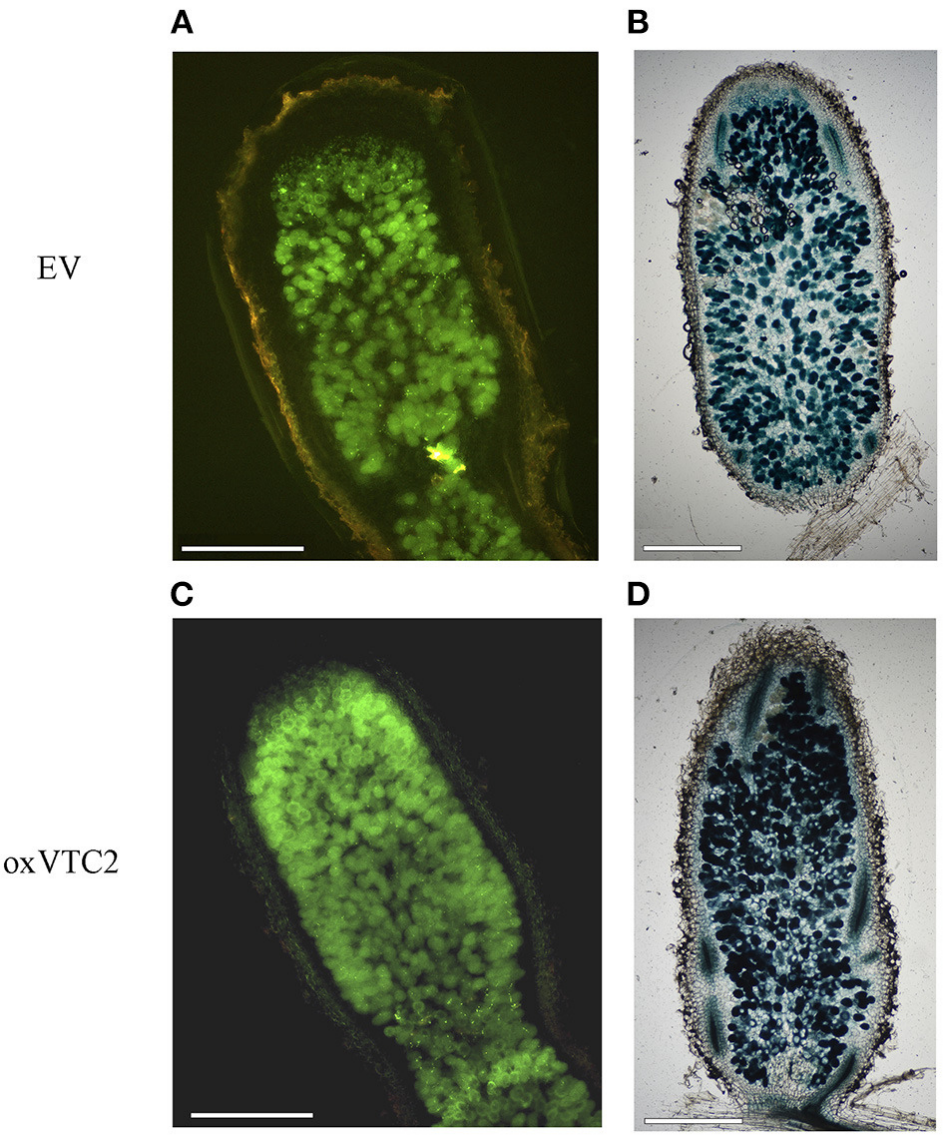
Representative images of *M. truncatula* root nodule sections of the EV **(A,B)** and oxVTC2 lines **(C,D)** upon inoculation with *E. meliloti* bacteria constitutively expressing GFP **(A,C)** or LacZ **(B,D)**. Six weeks after inoculation nodules were collected, sectioned (and stained in the case of LacZ), and visualized using a fluorescent or light microscopy. Selected images are representative images corresponding to observations carried out in two independent experiments. Scale bars represent 500 μm.

To test if increased biosynthesis of AsA could affect nodule development or the antioxidant status in nodules, we compared bacteroid occupancy in infected cells and accumulation of ROS in the two sets of lines. We used two *E. meliloti* 2011 strains constitutively expressing either GFP or LacZ and monitored the intensity levels of the fluorescent protein or the product of the X-Gal reaction in infected cells of the nitrogen-fixing zones of nodules (region FIII) using microscopy. Based on image analysis, nodule occupancy in the oxVTC2 lines was found comparable to those observed in EV nodules (Figure 2). These results were supported by the β-galactosidase activity assay in the nodule bacteroid fraction from both plant lines, with average activity of 18.77 ± 1.19 Miller units μg protein^−1^ in the EV line and 20.16 ± 4.18 Miller units μg protein^−1^ in the oxVTC2 line, respectively (*n* = 4 biological replicates per line).

Similarly, qualitative comparisons of the levels of H_2_O_2_ and ${\text{O}}_{2}^{-}$ did not appear to differ in nodules formed in the EV lines compared to the *MtVTC2*-overexpressing lines (Supplementary Figure 2).

### oxVTC2 *M. truncatula* Plants Were Unaltered in Drought Response

**Figure 3:**
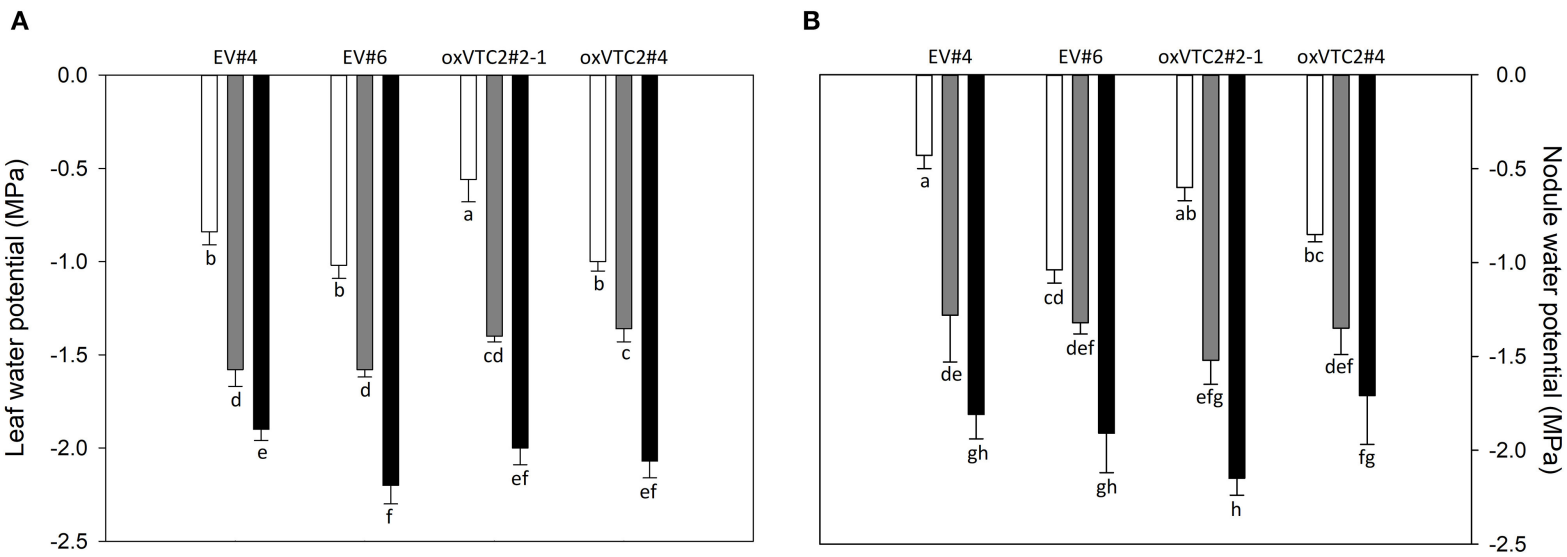
Effects of progressive drought stress on leaf **(A)** and nodule **(B)** water potentials in *M. truncatula* empty vector (EV) and oxVTC2 plants under well-watered control (C), mild drought (MD), and severe drought (SD) conditions. Values represent the average ± SE (*n* = 5 biological replicates). Mean values represented by the same letter do not differ statistically (*p* ≤ 0.05 ANOVA test).

**Figure 4:**
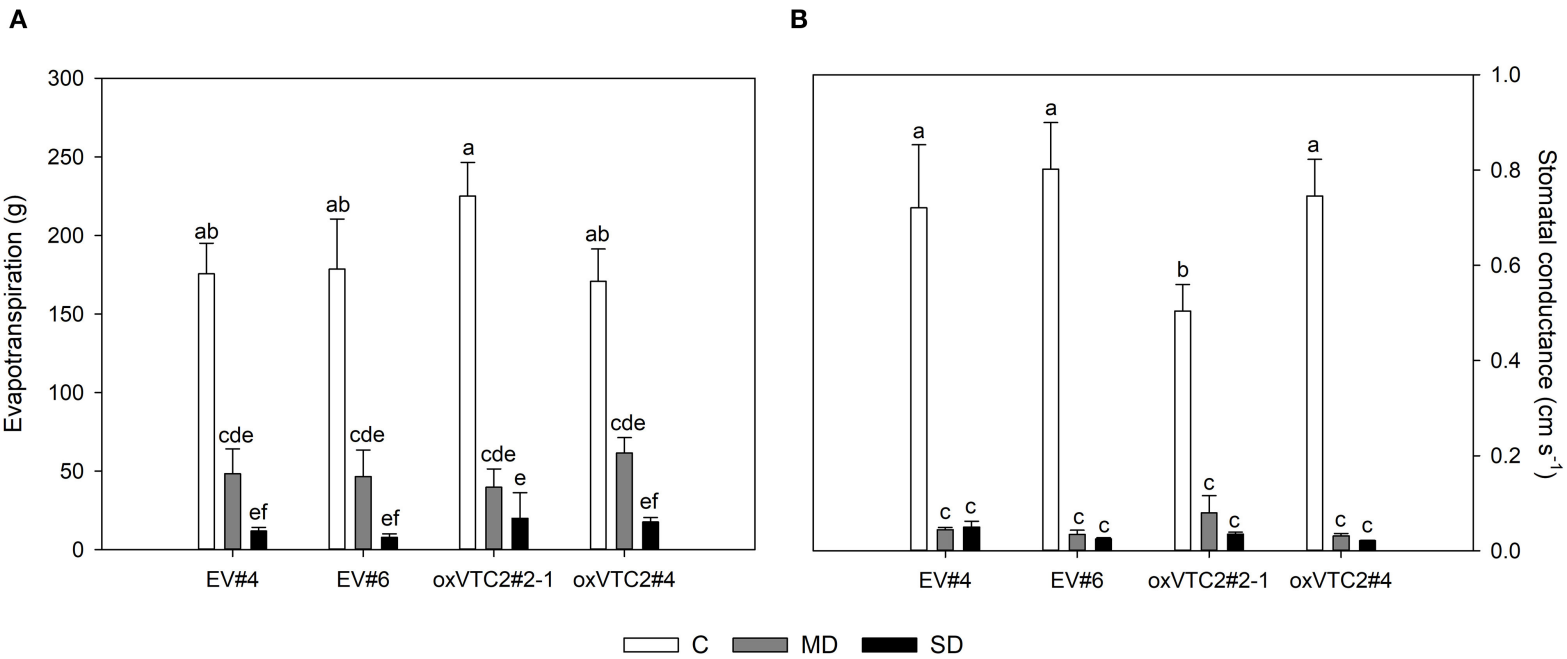
Effects of progressive drought stress on evapotranspiration **(A)** and stomatal conductance **(B)** in *M. truncatula* empty vector (EV) and oxVTC2 plants under well-watered control (C), mild drought (MD), and severe drought (SD) conditions. Values represent the average ± SE (*n* = 5 biological replicates). Mean values represented by the same letter do not differ statistically (*p* ≤ 0.05 ANOVA test).

To check whether the effect of increased AsA content in nodules could minimize the impact of water deficit in *M. truncatula*, we subjected the plants to progressive drought stress by withholding water for 6 days. Water potential in leaves and nodules, evapotranspiration and stomatal conductance were measured in plants following drought treatment. Both sets of plants showed a decline in leaf water potential levels reaching values around −1.5 and −2 MPa under mild drought (MD) and severe drought (SD) conditions, respectively (Figure 3A). A similar trend was observed for nodule water potential in the four lines tested (Figure 3B). Regarding evapotranspiration, under MD stress we observed a progressive decline in the levels of water lost in the EV and oxVTC2 lines (Figure 4A). Likewise, comparison of the two sets of plants revealed no significant differences in terms of stomatal conductance, with drought resulting in 95% decline in conductance regardless of the plant line (Figure 4B). The only exception was overexpressing line oxVTC2#2, which under control conditions showed relatively higher stomatal closure (Figure 4B). Likewise, plant biomass and chlorophyll levels did not show significant differences during the drought experiment regardless of the lines tested (data not shown).

**Figure 5:**
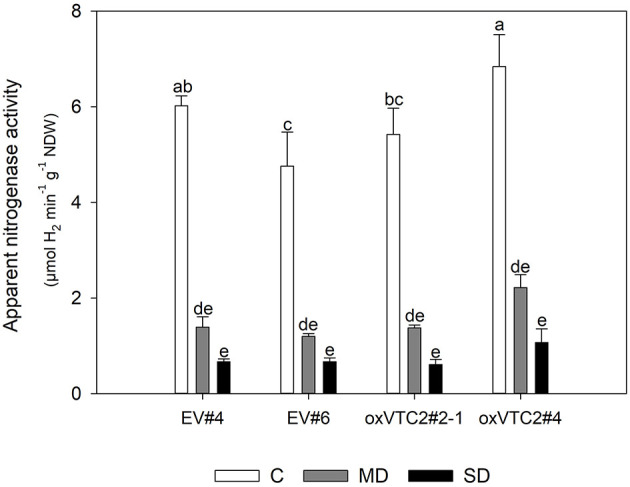
Effects of drought stress on nitrogen fixation in *M. truncatula* empty vector (EV) and oxVTC2 plants under well-watered control (C), mild drought (MD), and severe drought (SD) conditions. Values represent the average ± SE (*n* = 5 biological replicates). Mean values represented by the same letter do not differ statistically (*p* ≤ 0.05 ANOVA test). NDW, nodule dry weight.

Finally, to test if overexpression of *MtVTC2* could prevent drought-induced decline in nitrogen fixation, we measured ANA in the four lines. However, both the EV plants and the overexpression lines showed a similar reduction in the levels of ANA, showing no significant differences among genotypes (Figure 5). The inhibition of symbiotic nitrogen fixation before this of photosynthesis has been well-documented in the literature (Durand et al., 1987; González et al., 1995). Several studies in different plant species have shown that the decline in nitrogen fixation is not due to a limitation in carbohydrate transport to the nodule. In fact, the opposite is observed: sucrose, the main C compound transported to sink tissues, is accumulated in nodules during drought stress. To test if the decline in ANA levels in the present study was also associated to this regulatory mechanism, we measured the levels of sucrose in nodules of the EV and overexpressing lines (Supplementary Figure 3). Results show indeed a progressive accumulation of sucrose in nodules of both plant lines when subjected to water deficit conditions.

**Figure 6:**
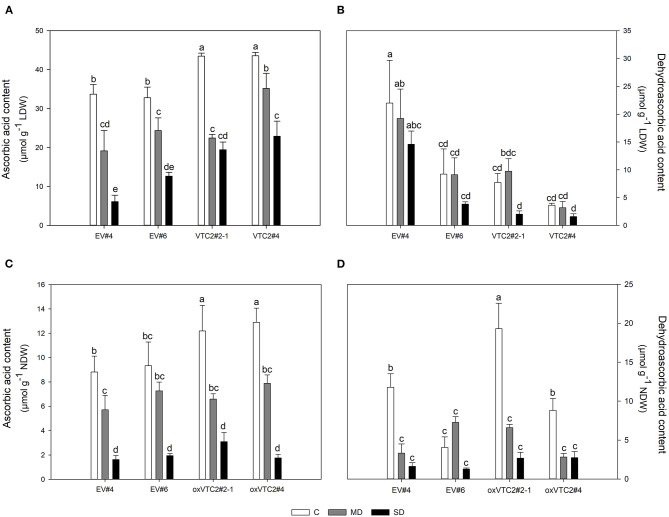
Levels of ascorbic acid (AsA) and dehydroascorbic acid (DHA) in leaves **(A,B)** and nodules **(C,D)** of *M. truncatula* empty vector (EV) and oxVTC2 plants under well-watered control (C), mild drought (MD), and severe drought (SD) conditions. Values represent the average ± SE (*n* = 5 biological replicates). Mean values represented by the same letter do not differ statistically (*p* ≤ 0.05 ANOVA test). LDW, leaf dry weight; NDW, nodule dry weight.

This drought-induced reduction in nitrogen fixation activity correlated well with the variations on the levels of AsA in leaves and nodules throughout the drought experiment (Figures 6A,C, respectively). In the EV lines, MD reduced the content of AsA in nodules by 30% in EV#4 while no significant effect was observed in EV#6 (Figure 6C). In contrast, SD had a similar impact on both EV lines reaching AsA values around 0.31 ± 0.03 mg g^−1^ NDW. Moderate levels of water deficit led to a 44 and 38% decline in AsA in lines oxVTC2#2-1 and oxVTC2#4, respectively (Figure 6C). As the stress increased, the content of AsA was reduced to 0.54 ± 0.13 and 0.31 ± 0.05 mg g^−1^ NDW in oxVTC2#2-1 and oxVTC2#4 lines, respectively. Regarding DHA, line oxVTC2#4 presented the highest DHA values in leaf tissue (Figure 6B), while in nodules oxVTC2#2-1 showed the highest DHA content under control conditions (Figure 6D). However, drought stress had a similar impact in all the lines tested, with a progressive decline as the water deficit became more severe in the case of leaves (Figure 6B), while the decline was much sharper in nodule tissue (Figure 6D). Interestingly, the only exception to this trend in nodules was line EV#6, which did not show significant variations in the content of DHA during the water deficit, most likely due to the relatively low levels of DHA detected in nodules from plants under control conditions (Figure 6D).

## Discussion

The enzyme VTC2, or GDP-L-galactose phosphorylase, is considered a control point of ascorbate biosynthesis in plants (Laing et al., 2007; Wolucka and Van Montagu, 2007). Increased expression of the genes coding for this enzyme in different plant species provide evidence for the critical role of this step in the pathway as a way to increase AsA content in plants (Bulley et al., 2009, 2012; Zhang et al., 2015).

Here we characterized the physiological responses of *M. truncatula* plants overexpressing *MtVTC2* under optimal water supply and when subjected to a progressive water deficit under symbiotic conditions. As AsA plays a significant role in ROS scavenging in nodules (Ross et al., 1999; Matamoros et al., 2006; Becana et al., 2010), it might be expected that increasing AsA biosynthesis in symbiotic root nodules would enhance nitrogen fixation and, as a result, increase plant growth. Both *MtVTC2* and *MtVTC5*, the two genes coding for GDP-L-galactose phosphorylases in the *M. truncatula* genome, are expressed in all plant organs analyzed, including nitrogen-fixing root nodules (Figure 1). Constitutive overexpression of *MtVTC2* indeed led to increased AsA content in nodules and this result correlated well with the levels of expression of the corresponding gene (Medtr5g093390), which showed a 3.8-fold increase in nodules when compared with EV plants as measured by qPCR analysis (Supplementary Figure 1). In contrast, the higher content of AsA in nodules did not lead to an increase in nodulation nor improved symbiotic nitrogen fixation rates, with both EV and oxVTC2 lines exhibiting similar values (Table 2). Using *E. meliloti* bacteria constitutively expressing LacZ, β-galactosidase activity assays in bacteroids isolated from mature nitrogen-fixing nodules did not show significant differences at the level of bacteroid occupancy between the lines (Figure 2). Accordingly, plants did not exhibit higher biomass when grown under exclusively symbiotic conditions (Table 2).

To explore whether the *M. truncatula* oxVTC2 lines might be more tolerant of stress, which generates ROS, plant responses to drought were measured. Previous work on the *VTC2* mutant of *A. thaliana, vtc1*, showed that it was more sensitive than the wild type to a number of abiotic stresses, including ozone and UV-B radiation (Conklin et al., 1996). Since then, a number of reports have described the potential advantage of bioengineering plants with increased AsA as a strategy to build drought tolerance (e.g., Bao et al., 2016 or Ma et al., 2014). Nevertheless, the experimental estimation of the stress itself and the level of tolerance is often not optimally determined in these works. Experiments aimed at the analysis of the effect of drought stress require the monitoring of physiological parameters such as the plant water potential in order to adequately gauge the degree of water deficit. Such physiological characterization of plants is, however, scarce in the literature, and reports on drought tolerance are often based on morphological aspects such as plant wilting (Bao et al., 2016), without taking into consideration variations in leaf area index or differential plant transpiration rates. Furthermore, the application of compounds such as polyethylene glycol is often equated with drought stress (as in Ma et al., 2014), whereas it should be rather seen as an osmotic contribution to the total water potential. As an example of this variability in experimental approaches, contrasting results regarding the effect of overexpressing *VTC2* on plant growth and stress tolerance have been reported. For instance, Zhang et al. (2015) described improved seedling biomass in salt-stressed rice plants overexpressing *A. thaliana VTC2*, while subsequent studies in rice did not show significant variations in terms of shoot biomass when overexpressing the kiwifruit ortholog (Ali et al., 2019) or the *OsVTC2* counterpart (Broad et al., 2020). Again, one important limitation of the conclusions reached by Zhang et al. (2015) is that biomass results were expressed on a fresh-weight basis instead of on a dry-weight basis, as mandatory when working with water-deficit stresses.

In the current work, the physiological characterization of plants showed that both the EV and oxVTC2 lines experienced a similar level of progressive water deficit based on the observed declines in leaf and nodule water potentials (Figure 3). Accordingly, plants rapidly showed a reduction in stomatal conductance, regardless of the overexpression of *MtVTC2*, despite the involvement of the AsA redox state in guard cell signaling (Chen and Gallie, 2004). Likewise, plant growth was arrested and SNF inhibited in both sets of plants as a consequence of the stress, suggesting that oxVTC2 plants did not present improved drought tolerance.

The fact that nitrogen-fixing legumes with increased antioxidant levels did not show improved plant performance or stress tolerance is disappointing, but not unusual. Previous studies in our laboratory showed that the exogenous application of galactono-1,4-lactone, the precursor for AsA biosynthesis, to nodulated pea plants did not alleviate the negative effect of drought stress either (Zabalza et al., 2008). Furthermore, *Medicago sativa* plants overexpressing mitochondrial Mn superoxide dismutase also failed to show improved nitrogenase activity or growth either under well-watered conditions or when plants were subjected to drought stress (Rubio et al., 2002). In contrast, positive results were obtained by El Msehli et al. (2011) by overexpressing the enzyme responsible for the biosynthesis of (homo)glutathione, gamma-glutamylcysteine synthetase, in *M. truncatula* nodules. This overexpression led to improved nitrogen fixation rates (estimated as ARA) and increased expression of a number of symbiotic markers in nodules such as leghemoglobin, although data on plant biomass was not provided. One of the reasons behind the relatively low success of such approaches is the fact that antioxidant compounds such as AsA are key metabolites subjected to a complex regulation and associated to a variety of metabolic pathways, even having regulatory roles on their own. Other factors such as the specific site of production, including subcellular localization, may also play a role in the success of this type of experiments. Additionally, when plants are forced to increase the levels of any regulatory metabolite, this often leads to the unwanted negative effects as a consequence of an impaired metabolism, including stunted plant growth and developmental issues.

The use of constitutive promoters such as the CaMV35S is a matter of debate in the symbiotic scientific community. Although constructs using this promoter are widely used for symbiotic functional analysis in legumes, Auriac and Timmers (2007) presented data suggesting that this promoter was inactive in the nodule meristem and infected cells of *Medicago truncatula* root nodules. Taking this observation into consideration and even if in the current work the CaMV35S may not drive homogeneous expression through the nodule, the promoter did lead to increased *MtVTC2* expression and AsA levels in the tissue. It would be of interest to test whether the use of alternative promoters that are specifically expressed in infected cells of the nitrogen-fixing zone of the nodule leads to a different symbiotic outcome.

In summary, our results show that *M. truncatula* plants with elevated AsA content can be generated through the overexpression of *MtVTC2*. However, this increased AsA availability does not provide an advantage in terms of plant growth or symbiotic performance either under well-watered conditions or in response to drought stress.

## Data Availability Statement

The original contributions presented in the study are included in the article/Supplementary Material, further inquiries can be directed to the corresponding author.

## Author Contributions

RH and MU generated the transgenic lines and performed initial experiments. DK performed preliminary screening of transgenic plants and provided qPCR data. RH, DD, and MU conceived the project and supervised preliminary screening of transgenic plants. LC-P performed drought experiments and carried out the physiological characterization of the lines. MR performed microscopy analysis and contributed to the interpretation of the data. EL and CA-I supervised the drought experiments. EL wrote the manuscript with input from all authors.

## Conflict of Interest

The authors declare that the research was conducted in the absence of any commercial or financial relationships that could be construed as a potential conflict of interest.

## Supplementary Material

Supplementary Figure 1 Relative expression of *MtVTC2* in nodules of the empty vector (EV) and oxVTC2 lines measured by qPCR using the reference gene PTB (Kakar et al., 2008) for normalization. Values represent the average ± SD fold-change of relative expression in the oxVTC2 lines compared to the EV plants calculated using the 2^−ΔΔCq^ method (Livak and Schmittgen, 2001) (*n* = 6, three biological and two technical replicates).

Supplementary Figure 2 Representative images of *M. truncatula* root nodule sections of the empty vector (EV, **A,B**) and oxVTC2 lines **(C,D)** after staining for the detection of superoxide using nitrobluetetrazolium **(A,C)** or H_2_O_2_ using 3,3′-diaminobenzidine-HCl **(B,D)**. Scale bars represent 500 μm.

Supplementary Figure 3 Levels of sucrose in nodules of *M. truncatula* empty vector (EV) and oxVTC2 plants under well-watered control (C), mild drought (MD), and severe drought (SD) conditions. Values represent the average ± SE (*n* = 5 biological replicates). Mean values represented by the same letter do not differ statistically (*p* ≤ 0.05 ANOVA test). NDW, nodule dry weight.

Supplementary Table 1 List of primers for used for qPCR analysis.

## Abbreviations

AsA: ascorbic acid
DHA: dehydroascorbate
Ψw: water potential
WC: water content
FW: fresh weight
DW: dry weight
C: control
SNF: symbiotic nitrogen fixation
ANA: apparent nitrogenase activity.
