# Supplementary figures and images for: Increased Ascorbate Biosynthesis Does Not Improve Nitrogen Fixation Nor Alleviate the Effect of Drought Stress in Nodulated Medicago truncatula Plants
Source: Front Plant Sci. 2021 Jun 28;12:686075. doi: 10.3389/fpls.2021.686075 (PMC8273863; doi:10.3389/fpls.2021.686075)

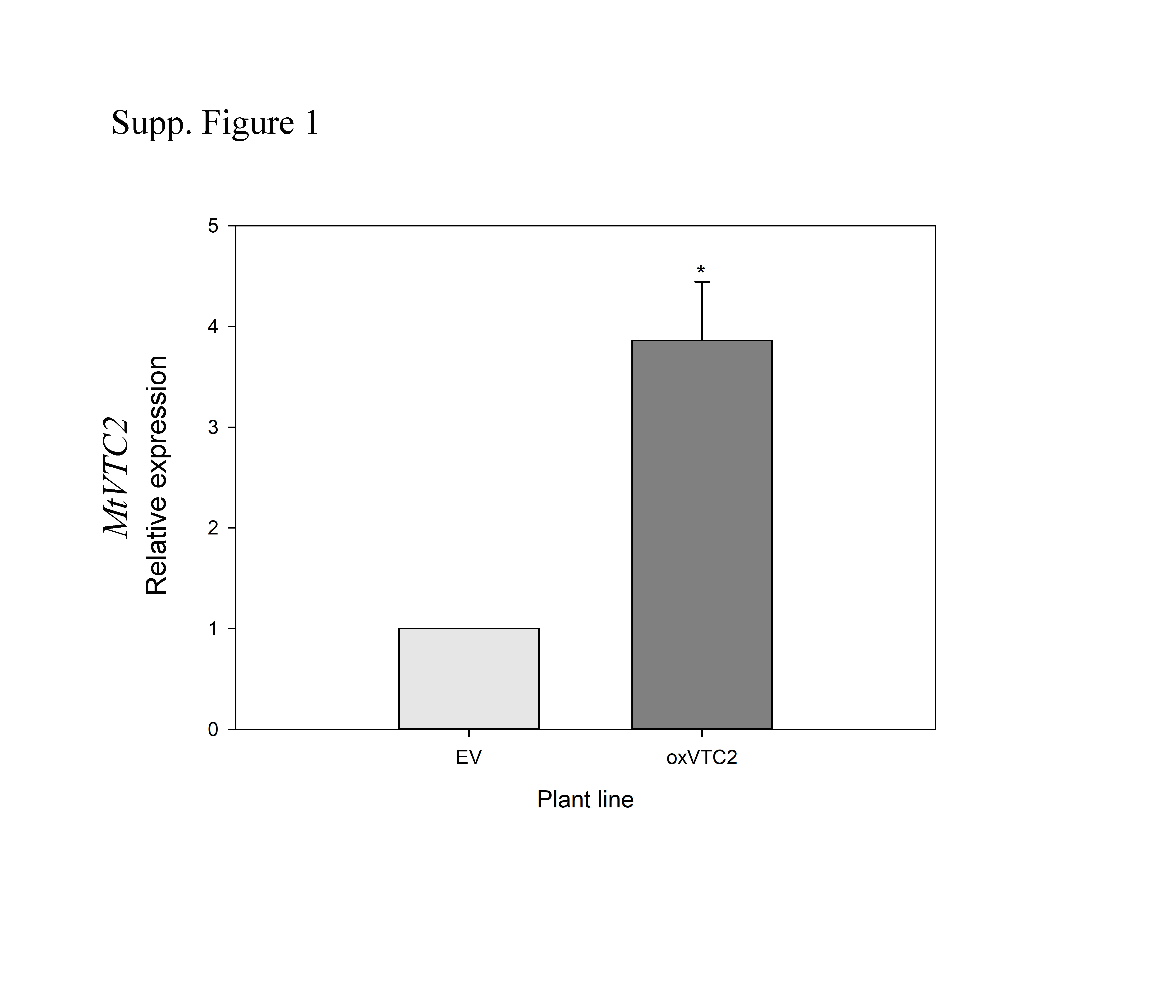

Supplement: Supplementary Figure 1 — Relative expression of MtVTC2 in nodules of the empty vector (EV) and oxVTC2 lines measured by qPCR using the reference gene PTB (Kakar et al., 2008) for normalization. Values represent the average ± SD fold-change of relative expression in the oxVTC2 lines compared to the EV plants calculated using the 2−ΔΔCq method (Livak and Schmittgen, 2001) (n = 6, three biological and two technical replicates). [file Image_1.JPEG]

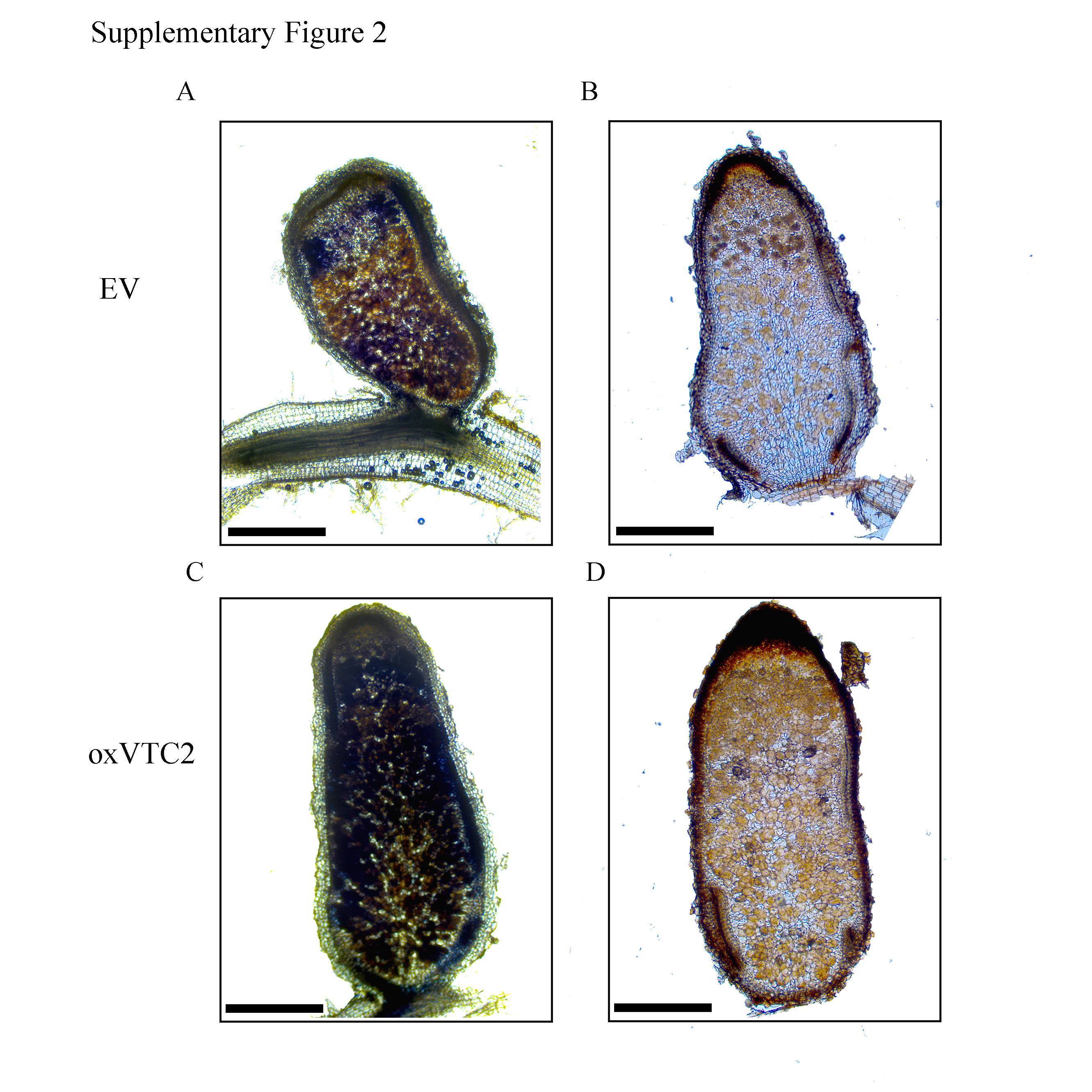

Supplement: Supplementary Figure 2 — Representative images of M. truncatula root nodule sections of the empty vector (EV, A,B) and oxVTC2 lines (C,D) after staining for the detection of superoxide using nitrobluetetrazolium (A,C) or H2O2 using 3,3′-diaminobenzidine-HCl (B,D). Scale bars represent 500 μm. [file Image_2.JPEG]

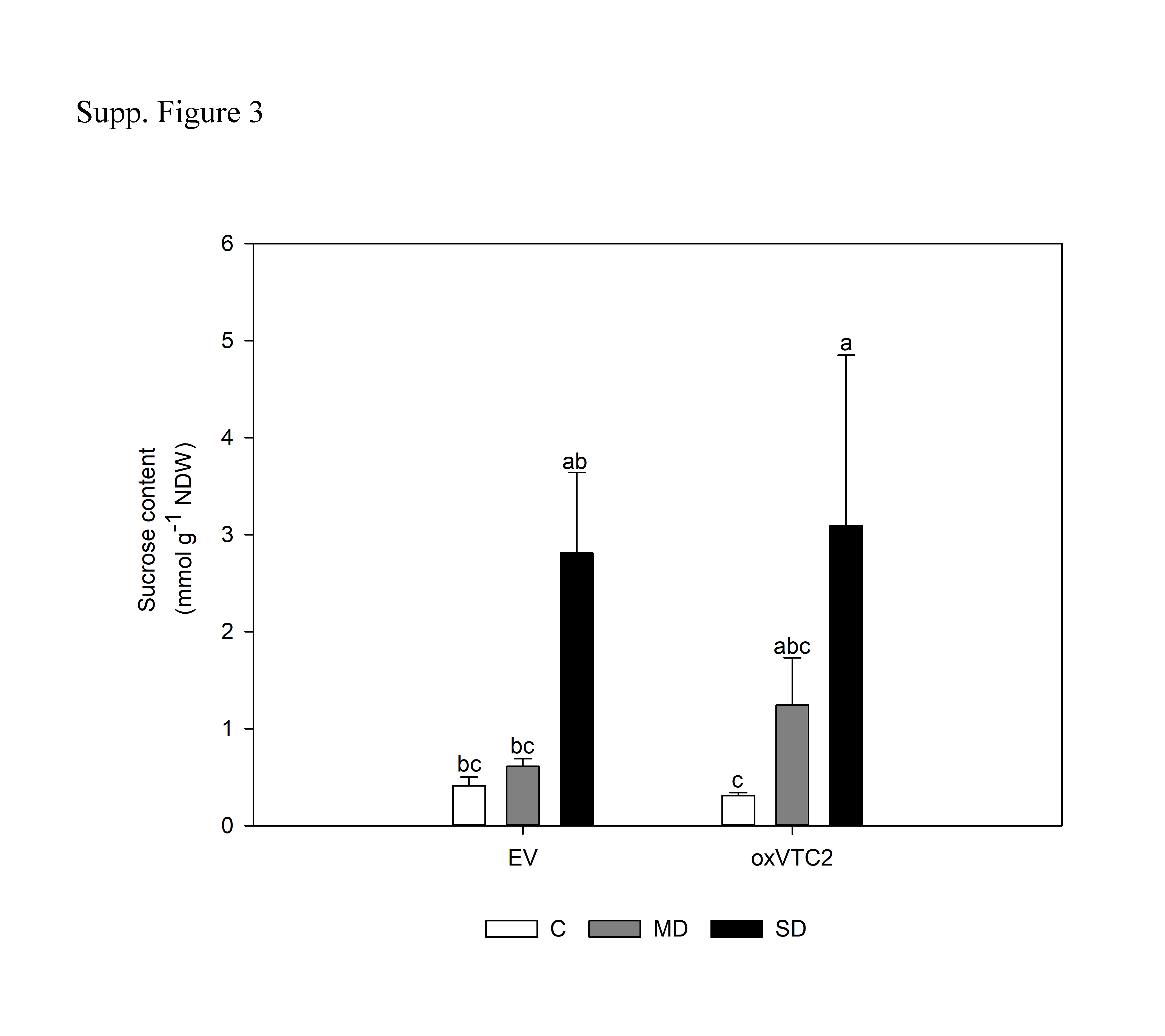

Supplement: Supplementary Figure 3 — Levels of sucrose in nodules of M. truncatula empty vector (EV) and oxVTC2 plants under well-watered control (C), mild drought (MD), and severe drought (SD) conditions. Values represent the average ± SE (n = 5 biological replicates). Mean values represented by the same letter do not differ statistically (p ≤ 0.05 ANOVA test). NDW, nodule dry weight. [file Image_3.JPEG]
